# Structural and thermal studies of silver nanoparticles and electrical transport study of their thin films

**DOI:** 10.1186/1556-276X-6-434

**Published:** 2011-06-22

**Authors:** Mohd Abdul Majeed Khan, Sushil Kumar, Maqusood Ahamed, Salman A Alrokayan, Mohammad Saleh AlSalhi

**Affiliations:** 1 King Abdullah Institute for Nanotechnology, King Saud University, Riyadh-11451, Saudi Arabia; 2 Department of Physics, Chaudhary Devi Lal University, Sirsa 125 055, India; 3 Department of Physics and Astronomy, King Saud University, Riyadh-11451, Saudi Arabia

**Keywords:** Silver nanoparticles, thin film, XRD, FESEM, FETEM, Electrical properties

## Abstract

This work reports the preparation and characterization of silver nanoparticles synthesized through wet chemical solution method and of silver films deposited by dip-coating method. X-ray diffraction (XRD), field emission scanning electron microscopy (FESEM), field emission transmission electron microscopy (FETEM), high-resolution transmission electron microscopy (HRTEM), selected area electron diffraction (SAED), and energy dispersive spectroscopy (EDX) have been used to characterize the prepared silver nanoparticles and thin film. The morphology and crystal structure of silver nanoparticles have been determined by FESEM, HRTEM, and FETEM. The average grain size of silver nanoparticles is found to be 17.5 nm. The peaks in XRD pattern are in good agreement with that of face-centered-cubic form of metallic silver. TGA/DTA results confirmed the weight loss and the exothermic reaction due to desorption of chemisorbed water. The temperature dependence of resistivity of silver thin film, determined in the temperature range of 100-300 K, exhibit semiconducting behavior of the sample. The sample shows the activated variable range hopping in the localized states near the Fermi level.

## Introduction

Metal nanoparticles with at least one dimension approximately 1-100 nm have received considerable attention in both scientific and technological areas due to their unique and unusual physico-chemical properties compared with that of bulk materials [1]. Phenomena at the nanoscale are likely to be a completely new world, where properties may not be predictable from those observed at large size scales, on account of quantum size effect and surface effects. Synthesis of nanoparticles has been a rapidly growing field in solid state chemistry [2]. Metal nanoparticles are particularly interesting because they can easily be synthesized and modified chemically as well as can suitably be applied for device fabrication [3-5]. Due to the specific size, shape, and distribution, nanoparticles are used in the production of novel systems such as nanosensors [6], nanoresonators [7], nanoactuators [8], nanoreactors [9], single electron tunneling devices [10], plasmonics [11], and nanowire based devices [12] etc.

Among the various metal nanostructures, noble metal nanoparticles have attracted much attention, due to their superior physical and chemical properties. Nowadays, a lot of researches have been focused on silver nanoparticles because of their important scientific and technological applications in color filters [13,14], optical switching [15], optical sensors [16,17], and especially in surface-enhanced Raman scattering [18-20]. Such properties and applications strongly depend on the morphology, crystal structure, and dimensions of silver nanostructures. Over recent years, silver thin films have been a subject of intensive investigations because of excellent optical, electrical, catalytic, sensing, and antibacterial properties [21,22] and subsequent applications. The synthesis of silver nanoparticles with controlled morphology is important for uncovering their specific properties and for achieving their practical applications.

Silver nanoparticles are of current importance because of its easy preparation process and unique optical, electrical, and thermal properties. The electrical conductivity of polyaniline-silver nanocomposite increases with increase in silver nanoparticles content than that of pure polyaniline [23,24]. Pillai et al. [25] demonstrated that solar cells employing metallic nanoparticles can dramatically enhance the near infrared absorption due to the presence of surface plasmons. The excited surface plasmons can eject electrons into a surrounding conductive medium resulting in effective charge separation. D. Basak et al. [26] observed significant modifications in the electrical properties of poly (methyl methacrylate) thin films upon dispersion of silver nanoparticles. So far as the electrical properties are concerned, it is necessary to throw some light on the structural and morphological characteristics of silver nanoparticles.

In the synthesis of nanoparticles, it is very important to control not only the particle size but also the particle shape and particle size distribution as well. In the present investigation, the synthesis of silver nanoparticles and thin films by wet chemical solution route [27] has been discussed. The prepared silver nanoparticles have been examined using X-ray diffraction (XRD), field emission transmission scanning electron microscope (FESEM), field emission transmission electron microscope (FETEM), high-resolution transmission electron microscope (HRTEM), two-probe direct-current (dc) resistivity measurement and thermogravimetric analysis/differential thermal analysis (TGA/DTA) thermal system.

### Experimental details

All the chemicals employed in the synthesis have been of analytical reagent grade. We used them without further purification. The nanoparticles of silver have been prepared according to the conventional procedure [28]. The aqueous solution (20 ml) containing glucose (9 mmol), polyvinylpyrrolidone (12 mmol), and sodium hydroxide (7 mmol) has been heated at 60°C for 30 min under vigorous stirring at 3,000 rpm. After that, 10 ml aqueous solution of AgNO_3 _(1 mol/l) has been dropped in the previous solution. After refluxing for 60 min, the colloidal solution has been allowed to cool slowly to room temperature. The resultant solution has been undertaken to centrifugation at 8,000 rpm for 90 min. After filtration, the precipitate so obtained has been washed many times with deionized water using centrifugation for 15 min each time. Finally, the precipitate has been collected and powdered finely, and identified as silver nanoparticles using characterization tools. These silver nanoparticles have been re-dispersed in ethanol for the preparation of silver film. The films have been deposited on ultra-clean quartz substrates using dip-coating method. The quartz substrate has been immersed vertically into the ethanol solution of silver nanoparticles (25 mg/ml). After that, the container has been placed in a vacuum chamber (10^-3 ^torr) at room temperature for 24 h; the smooth, uniform, and bright silver film has been obtained on the quartz substrate due to the evaporation of the solvent (ethanol) under reduced pressure. The film shows good adhesion to the substrate. Prior to the deposition of silver film, the quartz slide has been immersed in chromic-sulfuric acid for a day in order to clean the surface and to enhance its hydrophilicity; and then rinsed many times with deionized water and dried in air.

The morphology and crystal structure of silver nanoparticles powder has been evaluated by FESEM, FETEM, HRTEM, SAED, energy dispersive spectroscopy (EDX), and XRD. SEM images were obtained using a field emission scanning electron microscope (JSM-7600F, JEOL, Tokyo Japan) at an accelerating voltage of 15 kV. The fine powder of silver nanoparticles has been dispersed in ethanol on a carbon coated copper grid and TEM images were obtained with ultra-high resolution FETEM (JEOL, JEM-2100F) at an accelerating voltage of 200 kV. The reaction type and weight loss have been confirmed using TGA/DTA thermal system (DTG-60, Shimadzu, Kyoto, Japan). The XRD pattern was recorded by X-ray diffractometer (PANalytical X'Pert, Almelo, The Netherlands) equipped with Ni filter and CuKα (*λ *= 1.54056 Å) radiation source. For dc resistivity measurements, silver film with deposited contacts has been mounted in a specially designed metallic sample holder where a vacuum of about 10^-3 ^torr could be maintained throughout the measurements. A voltage (1.5 V, DC) was applied across the film and the resulting current was measured by a digital electrometer (Keithley 617, Keithley Instruments, Inc., Cleveland OH, USA). The temperature was measured by mounting a calibrated copper-constantan thermocouple near the sample.

## Results and discussion

### Structural properties

**Figure 1:**
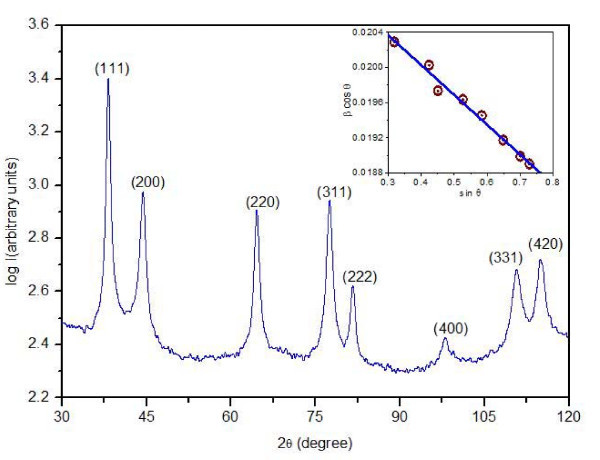
**XRD pattern of silver nanoparticles and inset shows Williamson-Hall plot for the same**.

Figure 1 shows the XRD pattern of powder silver nanoparticles. The presence of peaks at 2*θ *values 38.1°, 44.09°, 64.36°, 77.29°, 81.31°, 97.92°, 110.81° and 114.61° corresponds to (111), (200), (220), (311), (222), (400), (331), and (420) planes of silver, respectively. Thus, the XRD spectrum confirmed the crystalline structure of silver nanoparticles. No peaks of other impurity crystalline phases have been detected. All the peaks in XRD pattern can be readily indexed to a face-centered cubic structure of silver as per available literature (JCPDS, File No. 4-0783). The lattice constant calculated from this pattern has been found to be *a *= 0.4085 nm, which is consistent with the standard value a = 0.4086 nm. The crystallite size (*L*) of the material of thin film has been evaluated by Scherrer's formula [29]

where *λ *is wavelength (0.15418 Å) of X-rays used, *β *is broadening of diffraction line measured at half of its maximum intensity (in radian), and *θ *is Bragg's diffraction angle (in degree). The crystallite size of silver nanoparticles has been found to be 16.37 nm. In order to distinguish the effect of crystallite size induced broadening and strain induced broadening at FWHM of XRD profile, Williamson-Hall plot [30] has been drawn which is shown in inset of Figure 1. The crystallite size and strain can be obtained from the intercept at y-axis and the slope of line, respectively.

where *β *is FWHM in radian, *t *is the grain size in nm, *ε *is the strain, *λ *is X-ray wavelength in nanometers, and *C *is a correction factor taken as 0.94. The grain size and strain of the sample have been found to be 16.37 nm and 3.98 × 10^-3^, respectively.

The intrinsic stress (*σ*_s_) developed in nanoparticles due to the deviation of measured lattice constant of silver nanoparticles over the bulk has been calculated using the relation [31]

Here, *Y *is the Young's modulus of Ag (83 GPa), *a *is the lattice constant (in nanometers) measured from XRD data, *a*_0 _is the bulk lattice constant (0.5406 nm) and *γ *is the Poisson's ratio (0.37) for Ag.

The dislocation density (*δ*) in the nanoparticles has been determined using expression [32]

The X-ray line profile analysis has been was used to determine the intrinsic stress and dislocation density of silver nanoparticles and found to be as 0.275 GPa and 7.0 × ^-14 ^m^-2 ^respectively.

**Figure 2:**
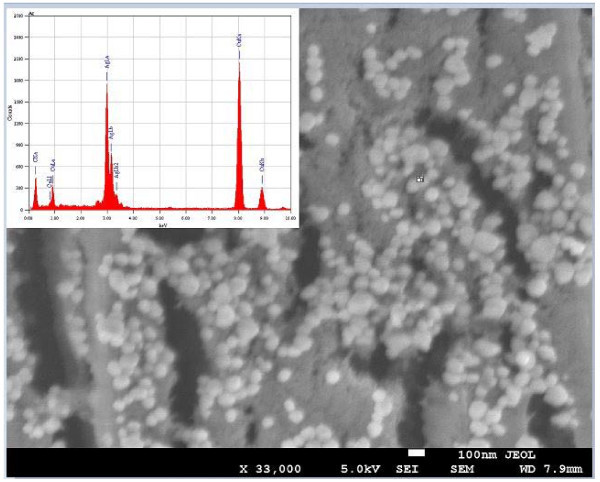
**FESEM image of silver nanoparticles and inset shows EDX profile for the same**.

**Figure 3:**
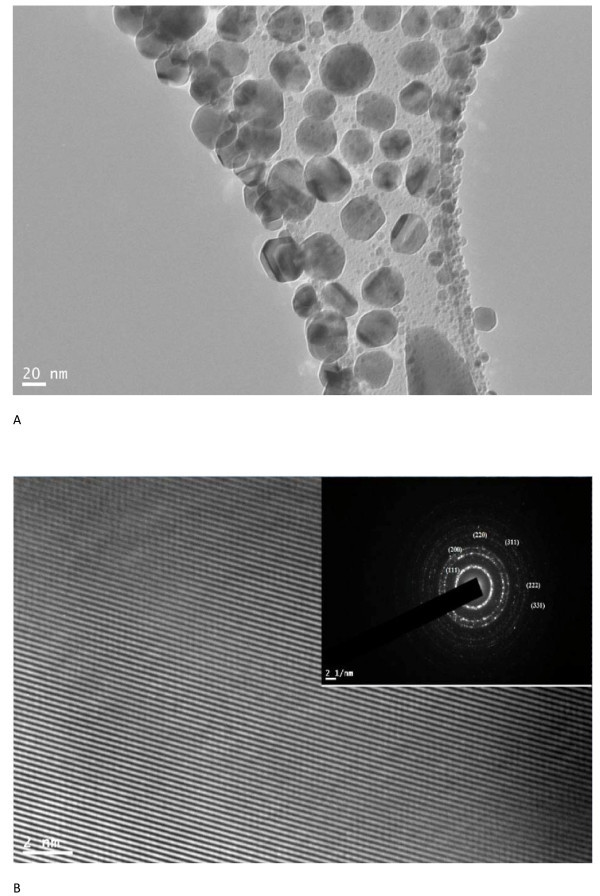
**FETEM and HRTEM images of silver nanoparticles**. (**a**) FETEM image of silver nanoparticles. (**b**) High-resolution image of a single silver nanoparticle and inset shows SAED pattern for the same.

Figure 2 shows the FESEM image of silver nanoparticles. It exhibits that almost all the nanoparticles are of spherical shape with no agglomeration. FETEM and HRTEM images of the same sample are shown in Figure 3a, b, respectively. Figure 3a shows that silver nanoparticles are spherical in shape having smooth surface and are well dispersed. The average diameter of silver nanoparticles is found to be approximately 35 nm. TEM image also shows that the produced nanoparticles have more or less narrow size distribution. HRTEM image (Figure 3b) has given us further insight into the microstructure and crystallinity of as-prepared silver nanoparticles. The clear and uniform lattice fringes confirmed that the spherical particles are highly crystallized. The lattice spacing of 0.232 nm corresponds to (111) planes of silver. The results show that the dominant faces of silver spheres are (111). The SAED pattern has been obtained by directing the electron beam perpendicular to one of the spheres. The hexagonal symmetry of diffraction spots pattern shown in the inset of Figure 3b confirmed that the spherical particles are well crystalline, and its face is indexed to (111) planes. Both HRTEM image and SAED pattern confirmed that the prepared spherical silver nanoparticles are single crystals.

The elemental analysis of sample has been performed using EDX spectroscopy. Inset of Figure 2 shows EDX spectrum of silver nanoparticles. The peaks observed at 3.0, 3.2, and 3.4 keV correspond to the binding energies of Ag *L_α_*, Ag *L_β_*, and Ag *L_β_*_2_respectively; while the peaks situated at the binding energies of 0.85, 1.0, 8.05, and 8.95 keV belong to Cu*L*_1_, Cu*L_α_*, Cu*K_α_*, and Cu*K_β_*, respectively. In addition, a peak at 0.25 keV corresponding to carbon (C*K_α_*) has been observed. The copper and the carbon peaks correspond to the carbon coated copper grid of TEM. No peaks of other impurity have been detected. Therefore, the EDX profile of sample (inset of Figure 2) indicates that the silver nanoparticles sample contain pure silver, with no oxide.

### Thermal properties

**Figure 4:**
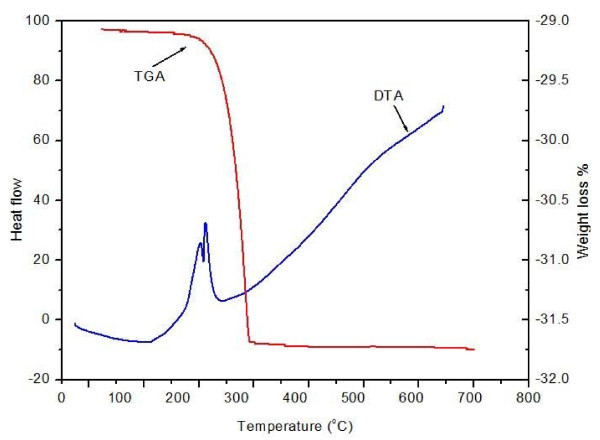
**DTA-TGA themogram of silver nanoparticles**.

TGA and DTA spectra have been recorded in temperature range from room temperature to 700°C using simultaneous thermal system (Shimadzu, DTG-60). A ceramic (Al_2_O_3_) crucible was used for heating and measurements were carried out in nitrogen atmosphere at the heating rate of 10°C/min. TGA and DTA curves of powder silver nanoparticles are given in Figure 4. It is observed from TGA curve that dominant weight loss of the sample occurred in temperature region between 200 and 300°C. There is almost no weight loss below 200°C and above 300°C. It can be generally attributed to the evaporation of water and organic components. Overall, TGA results show a loss of 14.58% upto 300°C. DTA plot displays an intense exothermic peak between 200°C and 300°C which mainly attributed to crystallization of silver nanoparticles. DTA profiles show that complete thermal decomposition and crystallization of the sample occur simultaneously.

### Electrical properties

**Figure 5:**
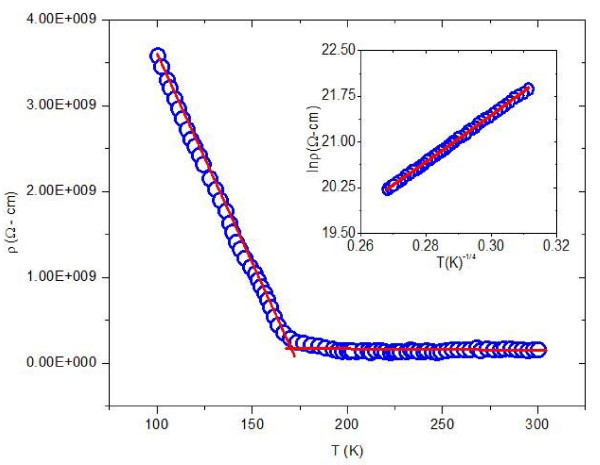
**Resistivity as function of temperature for thin film of silver nanoparticles**. Inset shows the plot of ln ρ vs T^-1/4^.

The temperature dependence of dc electrical resistivity of thin films of silver nanoparticles in the temperature range 100-300 K has been shown in Figure 5. It is evident from the figure that the resistivity decreases with increase in temperature, which shows the semiconducting nature of the sample. In these semiconductors, there are additional energy levels in the band gap, which are localized and close to either the conduction or the valence band. Since the energy difference between these levels and band edges is very small, a slight thermal excitation is sufficient to accept or donate electrons; thereby the electrical resistivity decreases with increase in temperature. Electron transport in the nanocrystalline silver thin film at relatively low temperature could be explained by thermally activated hopping between localized states near the Fermi level. In the variable range hopping (VRH) process [33], it becomes favorable for an electron to jump from one localized state to another where the overlapping of wave functions exists. The difference in corresponding eigen energies is compensated by the absorption or emission of phonons. Thus, the variation of electrical resistivity with temperature can be described by three-dimensional Mott's variable range hopping model [34],

where ρ_o _is the high temperature limit of resistivity (in *Ω*-*m*)and *T*_o _is Mott's characteristic temperature (in kelvin) associated with the degree of localization of the electronic wave function.

The Mott's characteristic temperature *T*_o _for three-dimensional hopping transport is given by,

where *k*_B _is the Boltzmann constant (in electronvolt per kelvin), *N*(*E*_F_) is the density of states (in per electron volt per cubic meter), 1/γ is the decay length of electronic wave function which typically varies in the range 3-30 Å and *C*_o _is a dimensionless constant, which has a value in the range 16-310 [35]. It is clear from Figure 5 and its inset that the sample exhibits a good fitting over the entire temperature range 100-300 K. Here, we have taken the localization length *γ *= 3 Å as reported by Maddison et al. [36]. From the fitted values of *T*_o_, we have found the value of density of states at the Fermi level *N*(*E*_F_) approximately 3.732 × 10^24 ^eV^-1 ^m^-3 ^for silver nanoparticles.

## Conclusions

The present wet chemical solution method for the preparation of silver nanoparticles and their thin films is simple, convenient, and viable which allows nanocrystalline silver particles of spherical shape and almost narrow size distribution. The x-ray diffraction pattern of sample shows a face-centered cubic crystalline phase of silver with lattice constant 0.4085 nm. The average particle size, as obtained from FETEM analysis, is 17.5 nm that agreed with XRD results. TGA/DTA study shows that the dominant weight loss occurs between 200°C and 300°C; and the reaction is of exothermic type. The temperature dependence of resistivity of silver film exhibits semiconducting behavior of the sample. The electrical conduction is due to the activated VRH in the localized states near the Fermi level.

## Competing interests

The authors declare that they have no competing interests.

## Authors' contributions

MAMK participated in the design of the study and performed the electrical studies. SK and MA carried out the structural studies. SAA and MSA performed the thermal studies. MAMK and MA also involved in writing of the manuscript. All authors read and approved the final manuscript.
